# Supplementary material for: Autonomous retrieval for continuous learning in associative memory networks
Source: Front Comput Neurosci. 2025 Aug 26;19:1655701. doi: 10.3389/fncom.2025.1655701 (PMC12418250; doi:10.3389/fncom.2025.1655701)
Supplement: Supplementary file 1 [file Table_1.pdf]

1 Appendix

1.1 Derivation of the gradient descent learning rule

Given the differential equation of a continuous Hopfield (CHN) network:

$$c\frac{du_i}{dt} = \sum_j W_{ij}v_j - \frac{u_i}{r} \tag{1}$$

At steady state, when $\frac{du_i}{dt} = 0$, from Eq. 1 we have:

$$u_i = r\sum_j W_{ij}v_j$$

where $v_j = \sigma(u_j)$. We can then define

$$\hat{u}_i = r\sum_j W_{ij}\sigma(\tilde{u}_j)$$

the expected potential of the unit i when the rest of the units are at their target potential $\tilde{u}_j$.

We can then define the error function E as the sum of squared differences between the expected potentials $\hat{u}_i$ and target potentials $\tilde{u}_i$:

$$E = \frac{1}{2}\sum_i(\tilde{u}_i - \hat{u}_i)^2$$

Taking the derivative with respect to the expected potential:

$$\frac{\partial E}{\partial \hat{u}_i} = (\tilde{u}_i - \hat{u}_i)$$

To compute how the error changes with respect to the weights W_{ij}, we use the chain rule:

$$\frac{\partial E}{\partial W_{ij}} = \frac{\partial E}{\partial \hat{u}_i}\frac{\partial \hat{u}_i}{\partial W_{ij}}$$

The partial derivative with respect to W_{ij} is:

$$\frac{\partial \hat{u}_i}{\partial W_{ij}} = rv_j$$

This is an approximation that captures the immediate direct effect of W_{ij} on $\hat{u}_i$, assuming that v_j remains constant and ignoring feedback effects in the recurrent network. This approximation is valid when the optimization steps are small relative to the nonlinearities in the sigmoid function.

Therefore, the error gradient with respect to W_{ij} becomes:

$$\frac{\partial E}{\partial W_{ij}} = (\tilde{u}_i - \hat{u}_i)rv_j$$

Finally, we update the weights using gradient descent:

$$\Delta W_{ij} = \alpha\frac{\partial E}{\partial W_{ij}} = \alpha r(\tilde{u}_i - \hat{u}_i)v_j$$

where α is the learning rate, typically set to 0.001 to ensure convergence.

1.2 Querying procedure

The network's ability to retrieve stored patterns can be tested through partial cues. Given a subset of informed units from a pattern μ, we initialize their potentials according to the partial pattern while leaving other units at rest :

$$u_i^\mu(t=0) = \begin{cases} +6 & \text{if unit } i \text{ is informed and } x_i^\mu = 1 \\ -6 & \text{if unit } i \text{ is informed and } x_i^\mu = 0 \\ 0 & \text{otherwise} \end{cases}$$

The network then evolves according to Eq. 1 until it reaches a stable state ($\|\frac{d\mathbf{u}}{dt}\|_\infty < \epsilon$). The thresholding procedure then allows for the reading of a stored binary pattern $\mathbf{x}^\mu$:

$$x_i^\mu = \begin{cases} 1 & \text{if } v_i(t_f) > 0.5 \\ 0 & \text{otherwise.} \end{cases}$$

with $v_i(t_f)$ the rate of neuron i after convergence of the dynamic. Alg. 1 summarize the procedure.

When operating below capacity, the dynamics typically converges to the stored pattern most similar to the initial cue. The final state is interpreted as a binary pattern using the thresholding procedure. As with traditional DHNs, retrieval success depends both on the network load and on the number of units informed in the initial cue. Although a theoretical analysis of the storage capacity under the GDA would be of interest, our focus here is on the autonomous retrieval mechanism, which operates in a regime well below this limit for which pattern retrieval is highly reliable.

Algorithm 1 Querying with convergence of the network using Euler method

```
Given a pattern μ and a subset of informed units
set u_i^μ(t = 0) = { +u_target   if x_i^μ = 1
                    { −u_target   if x_i^μ = 0
                    { 0           if x_i^μ not informed
repeat
    for each unit i do
        u_i(t+ = 1) = u_i(t) + δ(∑_j W_ij v_j − u_i(t)/r)
    end for
until ‖u̇‖_∞ < ε
define t_f = t                                  ▷ Final time at convergence
read : x_i^μ = { 1   if v_i(t_f) > 0.5
               { 0   otherwise.
```

1.3 Construction of correlated patterns

Algorithm 2 Generation of p correlated random patterns

```
Generate a random binary pattern x^{parent} ∈ {0,1}^N
Set k = ⌊(1 − ρ)N⌋, where ρ is the ratio of bits not randomized
for i = 1 to p do
    x^i ← x^{parent}
    Randomly select k distinct indices {j_1, ..., j_k} from {1, ..., N}
    for m = 1 to k do
        x^i_{j_m} ← random choice from {0,1} with equal probability
    end for
end for
```

1.4 Model with inhibitory matrix

To allow the sequential exploration of stored patterns a second approach have been tested. A set of plastic inhibitory synapses W'_{ij} has been added to the network dynamics :

$$c\frac{du_i}{dt} = \sum_j W_{ij}v_j - \sum_j W'_{ij}v_j - \frac{u_i}{r} \tag{2}$$

$$W'_{ij} \leftarrow W'_{ij} + \beta v_i(t_f)v_j(t_f) \tag{3}$$

with $v_i(t_f)$ the rate of neuron i after convergence of the dynamics. Self-inhibition $W'_{i,i}$ is set to 0 and undergoes no plasticity. A model combining self-inhibition and plastic inhibitory synapses has been tested and yields the same qualitative results. β represents the potentiation strength of the inhibitory synapses and is selected as a small value, typically below 0.05. The plasticity rule in Eq.3 is Hebbian.

We can observe that, similarly to adaptation, plastic synapses allow the retrieval of correlated memories Fig. 1 and 2. The potentiation strength β has to be weaker than that for adaptation, as a neuron is contacted by many inhibitory synapses. The retrieval dynamics are similar to the ones observed with adaptation in every aspect.

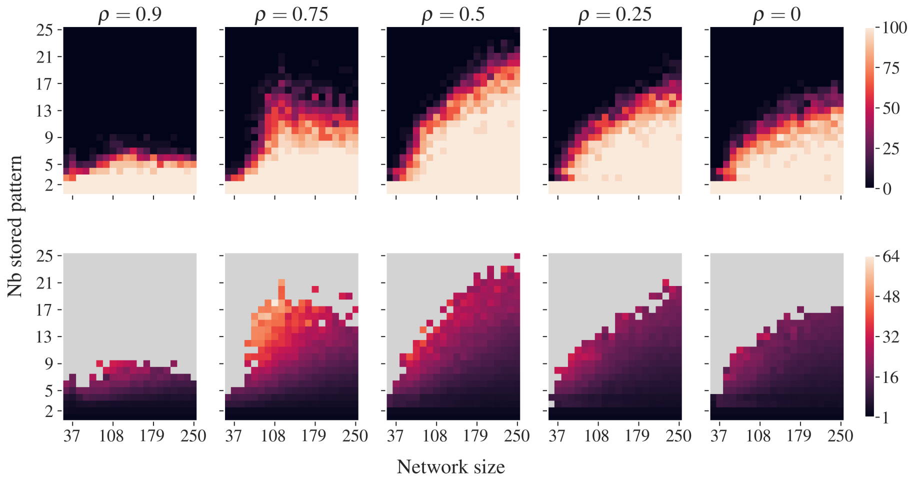

Fig 1. Recovery capacity of AR for various correlations but for a model using an inhibitory matrix $\mathbf{W}'$.

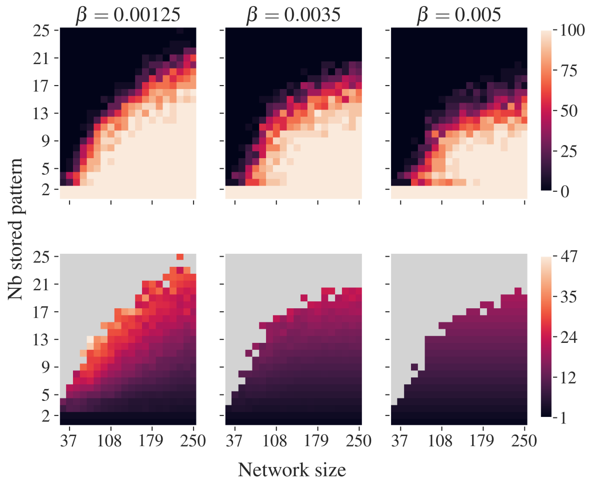

Fig 2. Recovery capacity of AR for various values of β but for a model using an inhibitory matrix $\mathbf{W}'$.

1.5 Drive-pattern correlation and spurious states

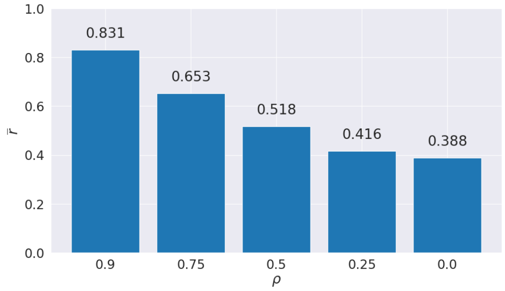

Fig 3. For networks trained with sets of varying correlation ρ, we compute the synaptic drive of each unit $I_j^{syn} = \sum_i W_{ij}$. This synaptic drive is the main component influencing the dynamic of the network at the neutral state, when the leak is null. Then, we compute the average correlation between each stored pattern and the synaptic drive: $\overline{r} = \frac{1}{p}\sum_{\mu=1}^{p} r^{\mu}$ with $r^{\mu} = \mathrm{Corr}(\mathbf{x}^{\mu}, \mathbf{I}^{syn})$. We can see that, the higher the ρ, the stronger the average correlation between the drive $\mathbf{I}^{syn}$ and stored patterns. At the neutral state, from which the network is initialized, stronger correlations induce a stronger push of the network state toward stored patterns. This could explain why our algorithm performs better with moderately correlated patterns.

1.6 Impact of sparsity on the recovery dynamics

Algorithm 3 Generation of p correlated sparse binary patterns

```
Input: sparsity s, correlation parameter ρ
Generate a binary parent pattern x^parent ∈ {0,1}^N with p(x_i = 0) = s
Set k = ⌊(1 − ρ)N⌋
for i = 1 to p do
    x^i ← x^parent
    Randomly select k distinct indices {j_1, ..., j_k} from {1, ..., N}
    for m = 1 to k do
        x^i_{j_m} ← 0 with probability s, otherwise 1
    end for
end for
```

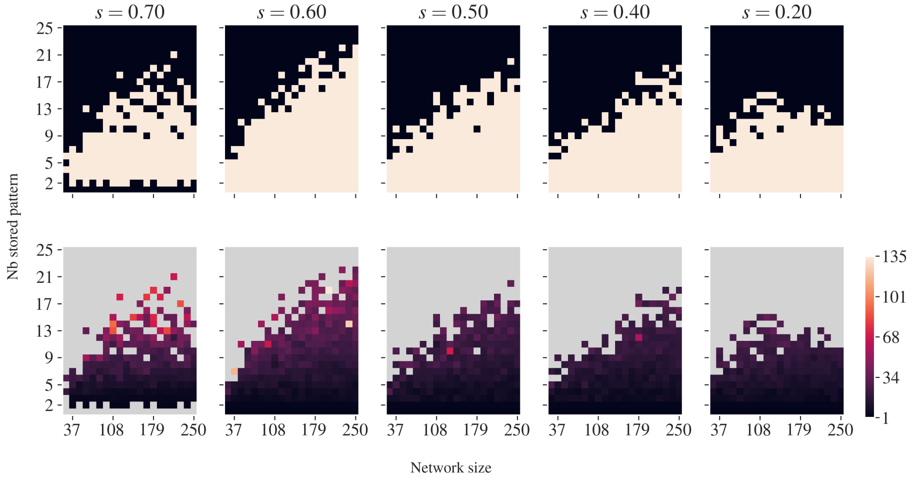

Fig 4. Recovery capacity of AR for various sparsity levels of the stored patterns. The data presented correspond to the results of AR for one simulation for each network size/number of stored patterns combination. **(Top row)** White pixels correspond to successful recovery of the full pattern set, while black pixels indicate the appearance of spurious patterns before full recovery is achieved. The appearance of spurious patterns for $s < 0.7$ with only one stored pattern is unexpected and necessitates further investigation.

1.7 Parameters for simulations

Table 1. CHN parameters

Name	Value	Description
ϵ_{sim}	10^{-6}	Convergence constant
r	1	Leak time constant
c	1	Integration time constant
δ	0.001	Euler step size

Table 2. Gradient descent

Name	Value	Description
α	0.0001	Learning rate
ϵ_{learn}	10^{-6}	Convergence constant
u^{target}	6	Target potential winning units
